# Cannabis-related information sources among US residents: A probability-weighted nationally representative survey

**DOI:** 10.1186/s42238-024-00249-5

**Published:** 2024-10-01

**Authors:** Kevin F. Boehnke, Tristin Smith, Michael R. Elliott, Adrianne R. Wilson-Poe, Daniel J. Kruger

**Affiliations:** 1 Chronic Pain and Fatigue Research Center, Anesthesiology Department, University of Michigan Medical School, 24 Frank Lloyd Wright Drive, Ann Arbor, MI 48106 USA; 2 Michigan Psychedelic Center, University of Michigan Medical School, Ann Arbor, MI USA; 3 Biostatistics Department, University of Michigan School of Public Health, Ann Arbor, MI USA; 4 Survey Research Center, Institute for Social Research, University of Michigan, Ann Arbor, MI USA; 5 Legacy Research Institute, Legacy Health, Portland, OR USA; 6 Jacobs School of Medicine and Biomedical Sciences, University at Buffalo, State University of New York, Buffalo, NY USA; 7 Department of Community Health and Health Behavior, University at Buffalo, State University of New York, Buffalo, NY USA

**Keywords:** Cannabis information, Survey, Healthcare provider

## Abstract

**Introduction:**

The Department of Health and Human Services recently recommended rescheduling cannabis from Schedule I to Schedule III, which might have broad effects on public health outcomes related to cannabis. In this changing environment, understanding national patterns in how people obtain information about cannabis is critical to informing public health outreach and education.

**Methods:**

We surveyed American adults (≥ 18 years) between June 22nd-26th, 2023 using the AmeriSpeak panel. We assessed past year cannabis use, intentions for cannabis use, and where participants got their information about cannabis. We investigated differences by past year use and explored associations between demographic and cannabis use characteristics with information sources using logistic regression.

**Results:**

Participants (*n* = 1,161) were 48.3±27.3 years of age (mean±standard deviation), 51% female, and 27% reported past year cannabis use. The most common information sources used were friends/family (35.6%) and websites (33.7%), while the least common information sources were health/medical care providers (9.3%), employees at place of purchase (8.6%), and government agencies (4.7%). Past year cannabis use was positively associated with all information sources except government agencies and popular media articles. A higher proportion of those using cannabis medically (with or without recreational use) obtained information from a healthcare provider (16.4% vs. 5.2%, *p* = 0.006).

**Conclusions:**

As cannabis accessibility increases and legality continues changing, there is a strong need for better clinician education, improved public health outreach, and improved communication between patients and clinicians about cannabis.

**Supplementary Information:**

The online version contains supplementary material available at 10.1186/s42238-024-00249-5.

## Introduction

In the past 30 years, cannabis has become increasingly accessible in the U.S., as 38 states and the District of Columbia have legalized medical cannabis as of April 2024 [1]. Although cannabis is federally classified as a Schedule I substance, designating it as a drug with no accepted medical use and a high potential for abuse [2], the Department of Health and Human Services recently recommended rescheduling cannabis from Schedule I to Schedule III [3]. This potential change in federal policy may have broad effects on the cannabis marketplace, how cannabis is used clinically, and public health outcomes related to changed cannabis legality. We have conducted large online surveys of naturalistic cannabis and cannabidiol (CBD) use showing that many individuals initiate cannabis use due to inadequate symptom relief, and that more individuals report that their rationale for use is personal research or a friend’s recommendation rather than recommendations from a healthcare professional [4, 5]. Further, a nationally representative survey conducted in 2017 showed that nearly half of the US population believe unsubstantiated claims about cannabis, such as that it is not at all addictive or that secondhand cannabis smoke is completely or somewhat safe [6]. Although these previous surveys investigate rationale for use and most influential information sources, little is known about the most common information sources from which people draw their cannabis-related information, which is important for informing public health outreach and education given the substantial societal impact of rescheduling cannabis to Schedule III. Based on previous surveys about cannabis information sources and known gaps in general knowledge related to cannabis [6–9], we hypothesized that participants would largely draw cannabis-related information from their own experiences and websites rather than healthcare providers.

## Methods

We surveyed American adults (≥ 18 years) between June 22nd-26th, 2023 using the National Opinion Research Center (NORC) AmeriSpeak panel. This probability-based panel of ~ 53,000 people is representative of 97% of American households, with a recruitment rate of 34% [10]. We assessed past year cannabis use (yes/no), reasons for cannabis use (medical only, non-medical only, both medical and non-medical), and where participants got their information about cannabis (Appendix). Information sources were drawn from previous research [4, 9, 11, 12] and included: *my own experimentation and experiences*,* a health/medical care provider*, *my medical cannabis caregiver*, *employees at place of purchase (e.g.*,* bud tenders)*, *friends and/or family*, *internet websites*, *government agencies*, *articles in the popular media (newspapers*,* magazines*,* etc.)*, *articles published in peer-reviewed scientific journals*, *some other source*, and *none of the above*. Note: Medical cannabis caregiver refers to an individual who is designated as a licensed provider to medical cannabis patients.

### Statistical analyses

We subgrouped by past-year cannabis use and intention for cannabis use. Because the medical only group was too small to be sufficiently powered for comparisons, we subgrouped by any medical use (medical only and combined medical and non-medical use) versus non-medical use only. We used χ2 tests to descriptively assess proportional differences in information sources by subgroups. Using logistic regression models, we investigated associations between demographic and cannabis use characteristics with information sources. Analyses included sampling weights such that estimates are representative of the U.S. population with respect to gender, age, education, race/ethnicity, and region. Analyses were performed in R.

### Ethics

This investigation was reviewed and approved by the NORC IRB and determined by the University of Michigan IRBMED to be an exempt study (Federal Exemption 2). We followed American Association for Public Opinion Research (AAPOR) reporting guidance for survey studies.

## Results

The survey was released to 6,666 panelists. Of these, 1,161 (17.5%) completed it, and the AAPOR Research Response Rate was 2.9%. Participants were 48.3±27.3 years of age (mean±standard deviation), 51% female, and 27% reported past year cannabis use (Table 1). The most common information sources used (Table 2) were friends/family (35.6%), websites (33.7%), and individual experimentation and experiences (22.0%). Conversely, the least common information sources were health/medical care providers (9.3%), employees at place of purchase (8.6%), government agencies (4.7%), and medical cannabis caregivers (4.4%). Participants with past year cannabis use were more likely to use all the information sources proposed -- except government agencies and popular media articles -- compared to people without past year cannabis use. Intention for cannabis use was associated with differential information sources, with a higher proportion of those using medically (with or without recreational use) obtaining information from a healthcare provider (16.4% vs. 5.2%, *p* = 0.006).

**Table 1:** Respondent characteristics by past year cannabis use (unweighted *n* = 1161)

	Total	Past Year Cannabis Use	No Past Year Cannabis Use	*p*-value
**Sex**				0.38
Males	614 (48.6%)	154 (45.6%)	460 (49.7%)	
Females	547 (51.4%)	136 (54.4%)	411 (50.3%)	
**Age (years)**				**< 0.001**
18–39	354 (37.3%)	120 (50.0%)	234 (32.7%)	
40+	807 (62.8%)	170 (50.0%)	637 (67.3%)	
Weighted Mean (SD)	48.3 (27.3)	41.8 (22.8)	50.6 (29.2)	**< 0.001**
**Race**				**<0.01**
Non-Hispanic White^a^	751 (61.7%)	169 (53.7%)	582 (64.5%)	
Non-Hispanic Black^ab^	150 (12.1%)	51 (19.6%)	99 (9.4%)	
Hispanic	182 (17.2%)	53 (19.9%)	129 (16.2%)	
Other^b^	78 (9.1%)	17 (6.9%)	61 (9.9%)	
**Income**				**< 0.01**
$0 - $49,999^a^	470 (39.9%)	140 (47.7%)	330 (37.0%)	
$50,000 - $99,999^b^	287 (33.3%)	90 (34.6%)	297 (32.8%)	
$100,000 + ^ab^	304 (26.9%)	60 (17.7%)	244 (30.2%)	
**Educational Attainment**				**< 0.01**
High School or Less^ab^	280 (37.9%)	84 (45.3%)	196 (35.2%)	
Some College/Associates Degree^a^	459 (26.4%)	117 (30.0%)	342 (25.1%)	
Bachelor’s Degree	255 (20.6%)	58 (15.5%)	197 (22.4%)	
Post Grad Study/Professional Degree^b^	167 (15.2%)	31 (9.3%)	136 (17.3%)	
**Cannabis Legal Status**				0.32
Recreational^a^	514 (40.6%)	146 (41.4%)	368 (40.3%)	
Medical Only^b^	321 (31.0%)	72 (26.5%)	249 (32.5%)	
Illicit^ab^	326 (28.6%)	72 (32.2%)	254 (27.2%)	

Data are reported as unweighted n (weighted percentages). Unweighted n for past year cannabis use is 1161, where 290 reported yes and 871 reported no. Participant responses were weighted such that estimates are representative of included states. Cannabis legal status refers to the current legality of cannabis dispensaries (recreational or medical) in a participant's state of residence. For categorical variables differences between groups assessed using chi-square tests. For continuous variables t-tests were used. ^a^and ^b^represent significant differences between groups on the pairwise level

**Table 2:** Participant-reported information sources for cannabis use by past year cannabis use and type of cannabis use

	Total (*n* = 1161)	Past Year Cannabis Use (*n* = 290)	No Past Year Cannabis Use (*n* = 871)	*p*-value	Total (*n* = 290)	Recreational Cannabis Use Only (*n* = 136)	Mixed or Medical Only Use (*n* = 154)	*p*-value
My Own Experimentation and Experiences	250 (22%)	165 (57.4%)	85 (9.1%)	**< 0.0001**	165 (57.4%)	80 (62.9%)	85 (53.1%)	0.2152
Health/Medical Care Provider	104 (9.3%)	38 (11.5%)	66 (8.5%)	0.2522	38 (11.5%)	9 (5.2%)	29 (16.4%)	**0.0064**
Employees at Place of Purchase	103 (8.6%)	77 (25.2%)	26 (2.6%)	**< 0.0001**	77 (25.2%)	29 (20.3%)	48 (29.1%)	0.2222
Friends and/or Family	401 (35.6%)	141 (45.9%)	260 (31.9%)	**0.0022**	141 (45.9%)	72 (54.3%)	69 (39.2%)	0.0675
Internet Websites	349 (33.7%)	117 (47%)	232 (28.8%)	**< 0.0001**	117 (47%)	53 (48%)	64 (46.3%)	0.8363
Articles in Popular Media	257 (21.8%)	50 (20.2%)	207 (22.3%)	0.6169	50 (20.2%)	26 (25.2%)	24 (16.3%)	0.2071
Articles Published in Peer-Reviewed Scientific Journals	112 (10.6%)	34 (14.4%)	78 (9.2%)	0.1016	34 (14.4%)	16 (18%)	18 (11.6%)	0.3318
Medical Cannabis Caregiver	40 (4.4%)	32 (13.4%)	8 (1.2%)	**< 0.0001**	32 (13.4%)	2 (1.4%)	30 (22.9%)	**< 0.0001**
Government Agency	61 (4.7%)	7 (3%)	54 (5.3%)	0.3295	7 (3%)	2 (1.3%)	5 (4.4%)	0.2094
Other Source	83 (8.2%)	23 (8.7%)	60 (7.9%)	0.7956	23 (8.7%)	10 (8.6%)	13 (8.8%)	0.9684
None of the Above	326 (28.4%)	23 (8.7%)	303 (35.4%)	**< 0.0001**	23 (8.7%)	13 (9.5%)	10 (8.1%)	0.7698

Data are reported as unweighted n (weighted percentages). Unweighted n for past year cannabis use is 1161, for type of cannabis use it is 290. Type of cannabis use refers to participant-reported use of cannabis (recreational, medical, or mixed use)

Past year cannabis use was positively associated with the following sources of information: Personal experimentation and experiences (AOR = 15.20; 95% CI [9.59, 24.09]), employees at the place of purchase (AOR = 15.42; 95% CI [8.02, 29.65]), friends and/or family (AOR = 1.90; 95% CI [1.28, 2.82]), internet websites (AOR = 2.21; 95% CI [1.50, 3.25]), and articles published in peer-reviewed scientific journals (AOR = 1.84; 95% CI [1.05, 3.22]) (Table 3). Further, women had higher odds of using friends and family as information sources compared to men (AOR = 1.81; 95% CI = [1.27, 2.59]). Compared to non-Hispanic White participants, those in other race/ethnicity groups had lower odds of utilizing their own experimentation and experiences (AOR = 0.54; 95% CI [0.34, 0.88]), friends and/or family (AOR = 0.62; 95% CI [0.41, 0.93]), and articles in popular media (AOR = 0.64; 95% CI [0.42, 0.97]). Of note, compared to those making <$50,000/year, those whose incomes exceeded $100,000/year had lower odds of relying on personal experimentation and experiences (AOR = 0.51; 95% CI [0.27, 0.97]). Higher income was also associated with higher odds of using articles published in peer-reviewed scientific journals ($50,000-$99,999: AOR = 2.34; 95% CI [1.14, 4.77]; $100,000 + per year: AOR = 2.82; 95% CI [1.36, 5.84]).

**Table 3:** Associations of demographic characteristics with participant-reported information sources of cannabis use

	My Own Experimentation and Experiences		Health/Medical Care Provider		Employees at Place of Purchase		Friends and/or Family		Internet Websites		Articles in Popular Media		Articles Published in Peer-Reviewed Scientific Journals	
**Variable**	**OR (95% CI)**	***p***-value	**OR (95% CI)**	***p***-value	**OR (95% CI)**	***p***-value	**OR (95% CI)**	***p***-value	**OR (95% CI)**	***p***-value	**OR (95% CI)**	***p***-value	**OR (95% CI)**	***p***-value
**Sex (ref. Male)**	0.67 (0.42, 1.07)	0.10	0.77 (0.44, 1.33)	0.34	0.77 (0.43, 1.39)	0.39	1.81 (1.27, 2.59)	**< 0.01**	0.94 (0.66, 1.33)	0.71	0.81 (0.55, 1.19)	0.27	1.29 (0.76, 2.19)	0.35
**Age (ref. 40 + years old)**	1.56 (0.96, 2.53)	0.07	0.79 (0.43, 1.44)	0.44	1.18 (0.65, 2.14)	0.58	1.35 (0.92, 1.98)	0.13	1.36 (0.93, 1.98)	0.11	0.71 (0.45, 1.12)	0.14	1.36 (0.79, 2.32)	0.27
**Race (ref. Non-Hispanic White)**														
Other Race/Ethnicity	0.54 (0.34, 0.88)	**0.01**	1.02 (0.57, 1.83)	0.95	1.39 (0.76, 2.54)	0.28	0.62 (0.41, 0.93)	**0.02**	1.23 (0.84, 1.78)	0.29	0.64 (0.42, 0.97)	**0.04**	0.86 (0.46, 1.6)	0.64
**Income (ref. $0 - $49**,**999)**														
$50,000 - $99,999	1.3 (0.74, 2.28)	0.37	0.94 (0.49, 1.79)	0.85	0.99 (0.48, 2.04)	0.98	1.57 (0.98, 2.53)	0.06	1.22 (0.78, 1.9)	0.38	0.99 (0.62, 1.56)	0.95	2.34 (1.14, 4.77)	**0.02**
$100,000+	0.51 (0.27, 0.97)	**0.04**	1.51 (0.76, 3.01)	0.24	1.51 (0.71, 3.21)	0.29	1.54 (0.93, 2.54)	0.09	1.1 (0.67, 1.8)	0.70	0.85 (0.51, 1.41)	0.52	2.82 (1.36, 5.84)	**< 0.01**
**Educational Attainment (ref. Bachelor’s Degree or Higher)**														
High School or Less	0.55 (0.30, 1.02)	0.06	1.5 (0.75, 3.00)	0.25	0.49 (0.23, 1.04)	0.06	0.91 (0.55, 1.52)	0.73	0.66 (0.40, 1.08)	0.10	0.9 (0.54, 1.52)	0.70	0.82 (0.42, 1.60)	0.56
Some College/Associates Degree	1.04 (0.57, 1.90)	0.90	1.79 (0.94, 3.42)	0.08	0.92 (0.47, 1.80)	0.80	0.94 (0.62, 1.42)	0.76	0.94 (0.62, 1.43)	0.78	1.21 (0.78, 1.86)	0.40	0.96 (0.53, 1.74)	0.89
**Cannabis Legal Status (ref. Illicit)**														
Recreational	0.73 (0.42, 1.27)	0.27	1 (0.53, 1.87)	0.99	2.71 (1.29, 5.70)	**< 0.01**	1.23 (0.82, 1.86)	0.32	1.06 (0.69, 1.63)	0.78	0.86 (0.54, 1.37)	0.51	1.27 (0.63, 2.55)	0.51
Medical Only	0.72 (0.40, 1.29)	0.26	1.57 (0.82, 2.99)	0.17	1.82 (0.75, 4.43)	0.19	0.84 (0.54, 1.31)	0.44	1.2 (0.76, 1.89)	0.43	0.68 (0.41, 1.11)	0.12	1.28 (0.62, 2.63)	0.50
**Past Year Cannabis Use (ref. No)**	15.2 (9.59, 24.09)	**< 0.0001**	1.49 (0.86, 2.60)	0.16	15.42 (8.02, 29.65)	**< 0.0001**	1.9 (1.28, 2.82)	**< 0.01**	2.21 (1.50, 3.25)	**< 0.0001**	0.96 (0.59, 1.56)	0.86	1.84 (1.05, 3.22)	**0.03**

Logistic regression models included all covariates in the same model

## Discussion

Results from our nationally representative survey show that most cannabis consumers obtain information about cannabis from friends or family, websites, and their own experiences, with very few obtaining information from medical or healthcare providers or government agencies. Even among the 173 participants using cannabis for medical purposes, only 16.3% reported obtaining information from a healthcare provider. Unsurprisingly, past-year cannabis use was most strongly associated with use of most information sources. Of note, higher income was associated with drawing information from peer-reviewed scientific articles and identifying as a race other than White was associated with lower odds of obtaining information from one’s own experimentation, friends/family, or articles in the popular media. Further qualitative research is needed to elucidate racial differences in information sources. Overall, these patterns are critical to understand for effective public health outreach strategies, especially given that the US Federal government may soon reschedule cannabis [3]. 

Because the majority of states have legal medical cannabis and nearly half of U.S. citizens live in states with legal adult-use cannabis [13], there is substantial need to improve cannabis-related public health outreach efforts. Practical education efforts should go beyond “abstinence only” messaging and focus on providing actionable advice on how to minimize harm and, if appropriate, maximize benefits of cannabis products. This could be done by briefly addressing content areas including routes of administration, differential effects of cannabinoids delta-9-tetrahydrocannabinol (THC) vs. CBD, dosing (“start low, go slow”), tolerance and side effects, as well as being explicit about understanding use intentions and goals to help enable mindful consumption [14–17]. Such efforts are critical to harmonize with education among healthcare providers. Indeed, a nationally representative survey conducted in 2017 showed that participants whose most influential cannabis-related information sources were health professionals and traditional media sources had lower odds of endorsing misinformation about cannabis than those who drew information from other sources, especially cannabis industry advertisements and social media platforms [9]. However, whereas clinicians frequently receive patient requests for medical cannabis authorization, many do not feel comfortable communicating about cannabis products, partially because they lack knowledge around medical effects, safety, and how to appropriately support patients using these products [18, 19]. Surveys and qualitative studies demonstrate that many physicians and medical students desire further relevant training (especially during medical school) [20–22], but only 9% of medical schools in 2016 offered medical cannabis-specific curricula [23]. As such, our findings suggest that in addition to conversations about cannabis occurring outside clinical settings, insufficient physician education may exacerbate misinformation about cannabis.

### Limitations and strengths

Our study has several limitations, including self-report and non-response bias, a fairly low response rate, and that we did not investigate how geographic variation affected responses. Further, we were unable to investigate how information sources used related to knowledge and understanding of the effects of cannabis products. Because we assessed information sources used rather than most influential or commonly used information source, we are unable to infer how these information sources rank in influencing decision making around cannabis. Additionally, we did not specifically delineate between internet websites and social media (the latter of is a major source of information for cannabis at present [9]), nor did we have explicit response options for television and radio. Although we did offer an “other source” option for people to fill in the blank, these limitations may have contributed to why 28.4% of participants reported “None of the above” for our information sources mentioned. It is also possible that there are other unmeasured sources of confounding that may influence which information sources people use, including but not limited to living in an urban versus rural area, previous legal issues related to cannabis (e.g., arrest or incarceration), and cultural factors. Although the Amerispeak panel implements best practices for probability-based recruitment, there may be unmeasured sampling biases [10]. Further, cannabis is still a stigmatized topic and self-reported data may be prone to social desirability bias. However, we minimized these risks by using a confidential survey design in which we only received de-identified data per NORC policies. Overall, these study limitations are also offset by our large sample size, minimal missing data (< 5%) and rigorous probability-weighted, nationally representative survey design.

## Conclusions

In this nationally representative survey, we show that most people draw information about cannabis from friends and family or online, with very few consulting their healthcare provider or government agencies. As cannabis accessibility and legality is increasing, there is a strong need for better clinician education, public outreach strategies, and improved communication between patients and clinicians about cannabis.

## Supplementary information

Supplementary Material 1

## Data Availability

Author Boehnke had full access to all of the data in the study and takes responsibility for the integrity of the data and the accuracy of the data analysis. *Concept and design: *Wilson-Poe, Elliott, Kruger, Boehnke. *Acquisition, analysis, or interpretation of data: *All authors. *Drafting of the manuscript: *Wilson-Poe, Smith, Boehnke. *Critical review of the manuscript for important intellectual content: *All authors. *Statistical analysis: *Smith, Elliott. *Obtained funding: *Boehnke. *Administrative, technical, or material support: *Kruger, Boehnke. *Supervision: *Boehnke.
