# Supplementary material for: Cannabis-related information sources among US residents: A probability-weighted nationally representative survey
Source: J Cannabis Res. 2024 Oct 1;6:38. doi: 10.1186/s42238-024-00249-5 (PMC11445971; doi:10.1186/s42238-024-00249-5)
Supplement: Supplementary file 1 — Supplementary Material 1 [file 42238_2024_249_MOESM1_ESM.docx]

## Appendix

## MI1.

Have you used cannabis (also known as marijuana) in the past year?

RESPONSE OPTIONS:

1. Yes
2. No

## MI2.

For what reasons have you used cannabis in the past year?

RESPONSE OPTIONS:

1. Only for medical reasons
2. Only for non-medical reasons, such as relaxation, socially, or recreationally
3. For both medical and non-medical reasons

## MI3.

In the past 30 days, how frequently have you used cannabis products of any kind (including smoking, eating, vaporizing, using topicals, or taking oils)?

RESPONSE OPTIONS:

1. Once
2. 2 to 3 times
3. Once a week
4. 2 to 3 days per week
5. 4 to 6 days per week
6. Once or twice a day
7. Three or more times per day

## MI4.

There are many compounds that come from cannabis. Have you heard of any of the following compounds?

1. Delta-8-THC
2. CBD
3. CBG
4. CBN

Response options for A-D

1. Yes
2. No

## MI5.

In the past 12 months, have you used any of the following compounds from cannabis?

1. Delta-8-THC
2. CBD
3. CBG
4. CBN

Response options for A-D

1. Yes
2. No

## MI6.

Where do you get your information about cannabis?

*Please select all that apply.*

RESPONSE OPTIONS:

1. My own experimentation and experiences
2. A health/medical care provider
3. My medical cannabis caregiver
4. Employees at place of purchase (e.g., bud tenders)
5. Friends and/or family
6. Internet websites
7. Government agencies
8. Articles in the popular media (newspapers, magazines, etc.)
9. Articles published in peer-reviewed scientific journals
10. Some other source
11. None of the above [SP]
